# Editorial: Processing technology for antioxidants in food production

**DOI:** 10.3389/fnut.2025.1576901

**Published:** 2025-03-06

**Authors:** Ziliang Liu, Jun Wang, Lizhen Deng, Chang Chen, Xianlong Yu

**Affiliations:** ^1^State Key Laboratory of Food Science and Resources, Nanchang University, Nanchang, China; ^2^College of Food Science and Engineering, Northwest A&F University, Yangling, China; ^3^Department of Food Science, Cornell AgriTech, Cornell University, Geneva, NY, United States; ^4^Shandong Academy of Agricultural Machinery Sciences, Jinan, China

**Keywords:** antioxidant materials, novel processing technology, quality detection, bioactive compounds, mechanism

The worldwide demand for nutritious foods are increasing with the global population growth. Natural antioxidant compounds, such as anthocyanins, phenolic, and flavonoids, are usually obtained from plant-based foods, which include fruits and vegetables, whole grains, nuts and seeds, and herbs. These antioxidant compounds exhibit numerous health benefits, such as preventing aging, reducing the risk of cardiovascular and neurodegenerative diseases, and inhibiting the growth of cancer cells.

Normally, antioxidant compounds are secondary metabolites generated by the plants as stress responses, and present in different forms in the plant matrix, including glycosidic and insoluble-bound, free and bound phenolics, and free forms. However, the antioxidant-containing foods are usually perishable due to the high water and nutrient contents. Therefore, various processing technologies, such as blanching, drying, freezing, and other physical/chemical pretreatment have been applied to extend their shelf life. It is widely agreed that there is hardly any processing technologies that can achieve maximum preservation of antioxidants and processing efficiency and throughput at the same time. Thermal treatment may result in the significant degradation of antioxidant compounds. Some non-thermal technologies including ultrasonic, freezing, and pulsed electric field could better preserve the antioxidants and facilitate the subsequent extraction yield, but they are subjected to relatively low processing efficiency and higher cost, particularly at large scale. Therefore, development of innovative technologies that leverage the processing efficiency, cost, and quality preservation of antioxidant-rich plant-based foods represent a critical need in the food industry and scientific research.

This Research Topic aims to promote the current and emerging processing technologies for preserving antioxidants in plant-based foods with high efficiency. Besides innovative processing technologies, this Research Topic also encourages the exploration of applying novel sensing and detection techniques in food processes to understand the processing mechanisms and impacts of food processing technologies on the antioxidant compounds in foods at macro-and micro-scale from mechanistic perspectives. Four research papers have been published in this Research Topic, which focus on new technologies and processes development, application, formulation and novel food development, which align well with the topic.

Li et al. investigated the effect of fermentation temperature on the non-volatile components and *in vitro* hypoglycemic activity of Jinxuan black tea. Thirty-six secondary metabolites were identified, including catechins, dimerized catechins, amino acids, flavonoid glycosides, phenolic acids, and alkaloids. Metabolomic study was performed to investigate the influence of fermentation temperature on the metabolite profiles. It was found that lower fermentation temperatures rendered higher polyphenol content and stronger hypoglycemic activity, which highlighted the importance of optimizing the fermentation temperature in enhancing the bioactive compounds and functionality of black tea.

Peterson et al. studied the “Impact of heat and high-moisture pH treatments on starch digestibility, phenolic composition, and cell bioactivity in sorghum (*Sorghum bicolor* L. Moench) flour.” It was revealed that wet-heating coupled with pH adjustment increased starch digestibility and preserved the phenolic content and bioactivity. This study underscored the potential of incorporating high-phenolic sorghum lines in future functional food formulations.

Awoyale et al. focused on studying the influence of added termite flour on the phytochemical properties of *Ogi* powder, and showed that addition of termite flour increased the contents of protein, crude fiber, ash, iron, and oxalate, but reduced the carbohydrates, zinc, phytate, and saponin contents. Proper ratio of *Ogi* powder and termite flour could give rise to a new *Ogi* powder product with better nutritional and phytochemical constituents as a nutrient supplement.

Zhu et al. designed a novel integrated infrared and heat pump drying technology for processing *Glycyrrhiza uralensis*. A computational fluid dynamic model was developed using the COMSOL software to simulate the air distribution within the dryer, and validated using experimental data on *Glycyrrhiza uralensis* drying. Under the optimized drying conditions, the retention of total flavone and total phenol contents in the *Glycyrrhiza uralensis* products was enhanced.

In summary, the published articles in this Research Topic have contributed to understanding the processing conditions and technologies including drying, fermentation, wet-heating, and modulation in treating antioxidant-rich plant-based foods during production. However, these studies are only the tip of the iceberg of innovative and emerging processing technologies. Further studies are encouraged and needed to enrich our knowledge base for designing, developing, and applying these technologies to improve antioxidant preservation, processing efficiency, and sustainability, which will contribute to an enhanced populational health and nutrition balance at regional and global levels. Particularly, several important and emerging research areas are identified and proposed here: (1) Mechanistic understanding of the processing influence on the antioxidant preservation in plant based foods from an interdisciplinary perspective; (2) Development and application of novel sensing techniques that can be integrated into current and future food processes for *in-situ* monitoring and precise food processing; (3) Exploration of emerging artificial intelligence and machine learning tools that can used for smart control and optimization of food processes for improved efficiency, sustainability, and antioxidant preservation.

